# New York Polyclinic

**Published:** 1890-07

**Authors:** 


### NEW YORK POLYCLINIC.

The New York Polyclinic (see ad. page 17) closes its Eighth regular session June 14, 1890. The Summer session opens June 16, '90, and closes September 13, '90. The Winter session of '90 and '91 will open September 15.

More than four hundred practitioners have matriculated for this year.

